# A novel homozygous *DST* variant causes hereditary sensory and autonomic neuropathy in a Pakistani family

**DOI:** 10.1038/s41439-025-00330-2

**Published:** 2025-11-18

**Authors:** Asad Munir, Helen Nabiryo Frederiksen, Fawad Ali, Sabawoon Shah, Abdur Rashid, Sergey Oreshkov, Kashif Khan, Muhammad Shahzeb, Inam Ullah, Hamid Ur Rahman, Mukhtar Ullah, Muhammad Ansar, Atta Ur Rehman

**Affiliations:** 1 Department of Zoology, Faculty of Biological and Health Sciences, Hazara University, Mansehra, Pakistan; 2 Department of Ophthalmology, University of Lausanne, Jules Gonin Eye Hospital, Fondation Asile Des Aveugles, Lausanne, Switzerland; 3 Department of Neurology, Saidu Group of Teaching Hospital, Saidu Sharif, Swat, Pakistan; 4 Department of Medicine, Luqman International Hospital, Swat, Pakistan; 5 Department of Medical Laboratory Technology, Kohat University of Science and Technology, Kohat, Pakistan; 6 Advanced Molecular Genetics and Genomics Disease Research and Treatment Centre, Dow University of Health Sciences, Sindh, Pakistan

**Keywords:** Genetic counselling, Genetics research

## Abstract

Hereditary sensory and autonomic neuropathy type 6 (HSAN-VI) is a rare autosomal recessive neurological disorder that affects fewer than 1 in 1,000,000 individuals worldwide and is characterized by neonatal hypotonia, respiratory and feeding difficulties, impaired motor development and autonomic abnormalities with highly variable age of onset and severity. Here we report a novel homozygous *DST* variant in association with HSAN-VI in two Pakistani siblings.

Hereditary sensory and autonomic neuropathies (HSANs) are a group of rare neurological diseases characterized by progressive sensory neuropathy with a variable degree of autonomic and motor involvement^1–3^. Patients with HSANs display distal sensory loss, painless injuries and skin ulcers. In addition, patients may experience neuropathic arthropathy, fractures, osteomyelitis and soft tissue infections that occasionally lead to amputation of the lower limbs^3^. HSANs overlap clinically with Charcot–Marie–Tooth syndrome due to the involvement of distal muscle weakness and wasting^4^. HSANs are classified into eight distinct types (I–VIII) based on their molecular etiologies, clinical presentation or inheritance patterns. To our knowledge, HSANs are associated with pathogenic variants in 15 known genes that follow both autosomal dominant and recessive inheritance patterns^5^.

*DST* (OMIM: 113810) encodes dystonin, a cytoskeletal-linker protein belonging to the plakin family of proteins. It possesses three major domains: a central plakin and spectrin repeat domain, a C-terminal microtubule-binding domain and an N-terminal domain^6,7^. By utilizing alternative promoters or exons, *DST* produces several isoforms that predominantly express in the nervous system, muscle and skin/epithelial tissues^8^. Pathogenic *DST* variants have been implicated in epidermolysis bullosa simplex and HSAN-VI. HSAN-VI (MIM: 614653) is mainly characterized by neonatal hypotonia, respiratory and feeding difficulties, impaired motor development and autonomic abnormalities with varying age of onset and severity^9^.

Here, we present a consanguineous Pakistani family in which two of the four children were affected by HSAN-VI (Fig. 1A). Of the four siblings—three brothers and one sister—two consecutive older siblings, a 12-year-old boy and a 10-year-old girl, were affected by HSAN-VI, whereas the two younger siblings, aged 8 and 5 years, were healthy (Fig. 1A). Both patients initially presented with complaints of progressive body weakness, cognitive decline and difficulty in walking since early childhood. Pediatric history revealed that both patients had normal birth. However, delayed milestones such as learning disability, lack of normal neurological development, and intellectual impairment were noted since birth. The parents reported no history of seizures. Clinical examination revealed that both patients were suffering from generalized body wasting with paucity of facial expressions, small chin, low-set ears, decreased corneal reflexes, absent pupillary reflex, high-arched palate, joint contracture, limited hip extension, decreased pain response/pain insensitivity and hypotonic with intact reflexes. X-ray radiographic examinations of the proband’s chest, lumbosacral and cervical regions revealed no bone deformities, and magnetic resonance imaging (MRI) spine and nerve conduction study (NCS) studies were both unremarkable. Noncontrast brain MRI showed posterior cortical atrophy, mild cerebellar vermis atrophy and left maxillary sinusitis (Fig. 1E). Based on the above clinical manifestations, a clinical diagnosis of HSAN was declared in both patients.

**Fig. 1:**
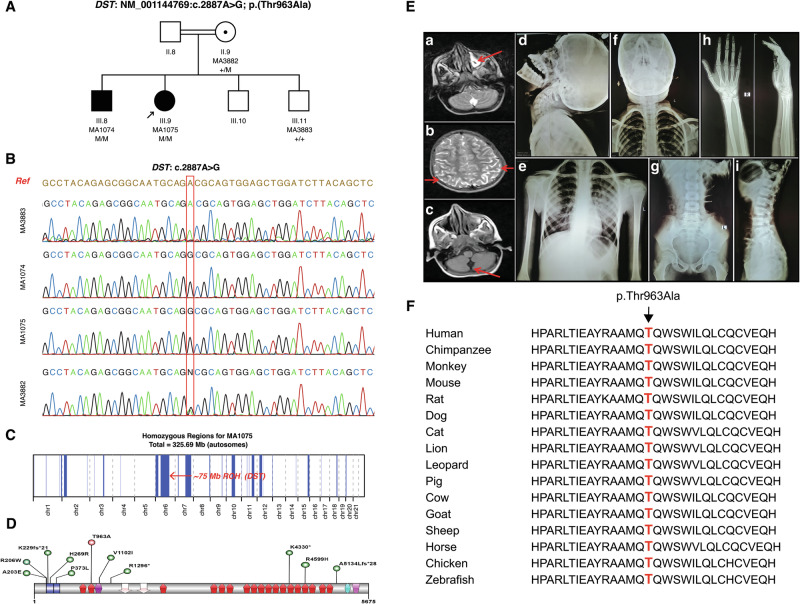
Clinical and genetic findings of the reported family. **A** Pedigree of the reported family showing parental consanguinity (+ indicates the wild-type allele, M indicates the identified variant and the arrow indicates the proband). **B** Chromatograms of genotyped individuals in the family (MA3883: healthy individual showing homozygous wild type nucleotide ‘A’; MA1074, MA1075: both patients showing homozygous mutant nucleotide ‘G’; MA3882: proband’s mother appearing as heterozygous carrier as confirmed through a double-peak in the chromatogram). **C** Homozygosity mapping shows chromosome-wide autozygous intervals as vertical blue peaks. The red arrow on chromosome number indicates the region of homozygosity (ROH) that harbors the *DST* gene. **D** Graphical display of published DST variants shown as vertical lollipops (green: previously published variants; red: variant identified in this study). The horizontal color scheme indicates different domains of DST protein (NP_001138241). The first and second calponin homology (CH) domains are shown as blue icons; the spectrin repeats (SPEC) as red icons; the SH3 domain as purple icons; the synaptonemal complex protein 1 (SCP-1) as baby-pink arrow icons; the EF-hand calcium-binding motif as sky-blue icons; and the Growth-Arrest-Specific Protein 2 (GAS2) domain as pink icons. **E** Red arrows on the MRI scan indicate (a) left maxillary sinusitis, (b) posterior cortical atrophy and (c) mild cerebellar vermis atrophy. X-ray radiographic examinations of the proband’s (d and e) cervical region lateral and AP, (f) right hand, (g and h) dorso-lumbosacral spine and (i) pelvis with both hip joints. All the X-ray radiographic examinations were unremarkable. **F** Multiple-sequence alignment indicating species-wise conservation of the altered residue (p.Thr963Ala).

For genetic diagnosis, we first extracted DNA from saliva samples obtained from available members of the family and then performed whole-exome sequencing (WES) on the proband’s DNA (ID: MA1075). Sequencing data from the proband were analyzed and annotated with reference to the GRCh38 human genome using an in-house developed computational pipeline. Region of homozygosity (ROH) were calculated from WES data using AutoMap^10^. WES data were manually curated for shortlisting high-quality and potentially disease-causing variants. This was done considering an autosomal recessive inheritance pattern and the relevance of the shortlisted gene or variant to the disease phenotype. VariantValidator was used for writing correct genomic nomenclature of the identified variant, whereas gnomAD, ClinVar, HGMD and Varsome were surveyed to gain insights into the deleteriousness of the identified variant. Sanger sequencing was used for variant validation as well as for genotype–phenotype cosegregation analysis. COBALT-NCBI was used to evaluate conservation of the altered residue across selected species.

WES identified a homozygous missense *DST* variant (NM_001144769.5:c.2887A>G; p.Thr963Ala) in the proband. The variant is ultrarare and has not been reported in population databases. Homozygosity mapping resulted in an accumulative 325.69-Mb (autosomal) genome in the proband to be homozygous, whereas the *DST* gene was flagged inside a large homozygous interval of ~75 Mb on chromosome 6 (Fig. 1C). These analyses reaffirm parental consanguinity (Fig. 1A) and provide modest support for the etiological role of the identified homozygous variant. In silico predictors mostly indicated that p.Thr963Ala is a damaging variant. The variant has a CADD-PHRED score of 27.1 and a PhyloP100 score of 6.179. Multiple sequence alignment confirmed that p.Thr963 is well conserved across vertebrate species (Fig. 1F). Familial cosegregation analysis revealed that both affected individuals were homozygous for the identified p.Thr963Ala variant, whereas their only available healthy brother was homozygous for the wild-type allele. Among the parents, the patient’s biological mother was a heterozygous carrier, whereas the father was unavailable for genotyping (Fig. 1A, B). Thus, consistent with pedigree-based inheritance pattern, and in line with published literature about *DST*, we recognize that the variant cosegregated recessively with the disease phenotype in the family. According to guidelines of the American College of Medical Genetics and Genomics, the identified variant was classified as likely pathogenic based on the criteria PM2_moderate, PM3_supporting, PP1_supporting, PP3_supporting and PP4_supporting.

*DST*-associated HSAN-VI is an ultrarare monogenic condition with a total of 16 patients across 7 families reported in the literature^5,11–16^. These include an Ashkenazi Jewish family^12^, two Italian families^11,14^ one Caucasian family^13^, one family from the USA^15^, one family from China^16^ and one family from Pakistan^5^. This means that our family is the world’s eighth case of HSAN-VI caused by a *DST* variant. In the Ashkenazi Jewish family, the disease phenotype was linked to a homozygous frameshift variant (p.Ala5134Leufs*28) whereas compound heterozygous variants (p.Arg1296* and p.His269Arg) were detected in the Caucasian family as well as in the two Italian families (p.Arg206Trp, p.Lys229fs*21 first family; and p.Lys4330*, p.Ala203Glu second family). With the addition of our variant (p.Thr963Ala) to the literature, the spectrum of HSAN-VI-causing *DST* variants now includes a total of 12 distinct alleles: 8 missense and 4 predicted loss-of-function variants (nonsense or frameshifts). Except one variant (NM_001374736:c.21899AG;p.Asp7300Gly), we have graphically represented all remaining 11 variants against the human DST (NP_001138241) protein as vertical lollipops. As shown in Fig. 1D, the majority of the previously reported variants are clustered toward the proximal region of the DST protein, highlighting its likely functional importance.

Although largely similar to the published reports, we have observed a slight variation in the clinical presentation of our patients. For instance, both patients in our study appeared to have delayed milestones, learning disabilities and mental retardation, whereas the proband’s brain MRI revealed posterior cortical atrophy, mild cerebellar vermis atrophy and left maxillary sinusitis. To the best of our knowledge, these symptoms have not been reported previously. Thus, our data add interesting insights into the clinical spectrum of *DST*-associated HSNA-VI as previously anticipated^6^. Notably, our patients lacked certain characteristic clinical features, including respiratory and feeding difficulties, delayed motor development, labile cardiovascular function and areflexia. However, given the progressive nature of HSAN-VI, it is likely that these symptoms may develop later in life. Although no concrete evidence currently explains the phenotypic heterogeneity observed in published cases, it has been speculated that protein-truncating variants probably cause loss of function in the neuronal isoforms, potentially leading to a more severe disorder characterized by congenital defects and early lethality. Meanwhile, missense *DST* variants probably retain partial protein function, resulting in milder phenotypes^11–14^. Interestingly, it has been found that restoring dystonin-a2 expression in neuronal tissues of an HSAN-VI mouse model attenuates the degeneration of sensory neurons, decreases disease severity and increases mice life span^17^.

In conclusion, we report clinical and genetic data from a consanguineous Pakistani family in which two children were affected by the rare neurological disorder HSAN-VI. We identified a likely pathogenic *DST* variant (p.Thr963Ala) as the underlying cause of the disease in the family. Overall, our patients represent the world’s eighth reported case of DST-associated HSAN-VI and the second from Pakistan to be documented in the literature.

## HGV Database

The relevant data from this Data Report are hosted at the Human Genome Variation Database at 10.6084/m9.figshare.hgv.3559.

## Data Availability

All the data obtained in this study are provided in this report.
